# Conditionally unbiased estimation in the normal setting with unknown variances

**DOI:** 10.1080/03610926.2017.1417429

**Published:** 2018-01-05

**Authors:** David S. Robertson, Ekkehard Glimm

**Affiliations:** a MRC Biostatistics Unit, University of Cambridge, Cambridge, UK; b Novartis Pharma AG, Novartis Campus, Basel, Switzerland; c Medical Faculty, Institute for Biometrics and Medical Informatics, Otto-von-Guericke-University Magdeburg, Magdeburg, Germany

**Keywords:** Selection bias, Two-stage sample, Uniformly minimum variance conditionally unbiased estimation, 62-07

## Abstract

To efficiently and completely correct for selection bias in adaptive two-stage trials, uniformly minimum variance conditionally unbiased estimators (UMVCUEs) have been derived for trial designs with normally distributed data. However, a common assumption is that the variances are known exactly, which is unlikely to be the case in practice. We extend the work of Cohen and Sackrowitz (*Statistics & Probability Letters*, 8(3):273-278, 1989), who proposed an UMVCUE for the best performing candidate in the normal setting with a common *unknown* variance. Our extension allows for multiple selected candidates, as well as unequal stage one and two sample sizes.

## 1. Introduction

Two-stage adaptive trial designs offer an efficient way of selecting and validating multiple candidate treatments within a single trial. A common strategy is to select the best performing treatment (according to some ranking criteria) after an interim analysis, and to then validate its properties in an independent sample in stage 2.

However, selecting and ranking candidates in this way can induce bias into the naïve estimates that combine data from both stages. If the selection rules are not properly taken into account by the estimation strategy, then intuitively one might expect overly optimistic estimates of the performance of the selected candidate, given that it had to perform ‘well’ in stage 1 in order to proceed to stage 2.

In order to efficiently and completely correct for this selection bias, the technique of Rao-Blackwellisation can be used, where the unbiased stage 2 data is conditioned on a complete, sufficient statistic. The resulting estimator is the uniformly minimum variance conditionally unbiased estimator (UMVCUE). An appealing feature of the UMVCUE is that, as the name suggests, it has the smallest variance (or equivalently, mean squared error (MSE)) amongst all possible unbiased estimators.

This two-stage estimation framework was introduced by Cohen and Sackrowitz (1989), who derived the UMVCUE for normally distributed data. Their work has since been extended to a variety of other trial settings with normal (or asymptotically normal) data. Bowden and Glimm (2008) extended the UMVCUE to apply to unequal stage one and two sample sizes, and when the parameter of interest belongs to the *j*-th best candidate out of *k*. In related work, Kimani, Todd, and Stallard (2013) derived UMVCUEs for the means of the selected and control treatments in the seamless phase II/III trial setting with early stopping. Meanwhile, Bowden and Dudbridge (2009) derived the UMVCUE for a two-stage genome-wide association study with ranking based on *p*-values. In a recent development, Robertson, Prevost, and Bowden (2016a, 2016b) generalised all of these approaches to normal data with an arbitrary correlation structure.

However, apart from the original Cohen and Sackrowitz paper, all of the above analyses assume that the variances of the parameter of interest are known exactly. This may not be a reasonable assumption to make. In practice, the variance of a treatment effect (say) will often be estimated directly from the data of the trial itself. Alternatively, the variance will be based on the results from a previous trial or pilot study on the same treatments.

Cohen and Sackrowitz did derive the UMVCUE for the mean of the selected normal population with a common unknown variance. However, the estimator is only valid for the highest ranked population when the stage 1 sample sizes are all equal, and the stage 2 sample size is equal to one. In this paper, we aim to address these limitations.

In Section 2 we present the model framework and the (corrected) form of the UMVCUE given by Cohen and Sackrowitz (1989). We extend their approach in Section 3, and in Section 4 compare the resulting UMVCUE with the one derived by Bowden and Glimm (2008) that assumes a known variance. A case study based on the INHANCE trial is presented in Section 5, and we conclude with a discussion in Section 6.

## 2. Equal sample sizes

To start with, we consider the setting of Cohen and Sackrowitz (1989) with a common *unknown* variance. Suppose there are *k* experimental treatments, with each tested on *n* subjects in stage 1. Let the stage 1 data *X_ij_*, *i* = 1, 2, …, *k*; *j* = 1, 2, …, *n*, be normally distributed with means μ_*i*_ and common unknown variance σ^2^. Denote the stage 1 sample means by ${\bar{X}}_{1},{\bar{X}}_{2},\ldots ,{\bar{X}}_{k}$.

At the end of stage 1, the treatment with the largest sample mean is selected for confirmatory analysis in stage 2. Let *Y* be a single observation taken from the highest ranked treatment group in stage 2. Also let *Q* be the event $\left\{X:{\bar{X}}_{1}>{\bar{X}}_{2}>\cdots >{\bar{X}}_{k}\right\}$. Without loss of generality, we condition on *Q*, as this can be viewed as simply a relabelling of the treatments.

Cohen and Sackrowitz derived the following UMVCUE for the mean μ_1_ of the highest ranked treatment:

Theorem 2.1. The UMVCUE of μ_1_ given *Q* is

(1) $$\frac{Z}{n+1}-\sqrt{\frac{n}{n+1}}\times \frac{\tilde{S}{\left(1-{\underline{r}}^{2}\right)}^{c}}{2^{2c}cB\left(c,c\right)F_{\beta (c,c)}\;\left(\frac{1}{2}\left(\underline{r}+1\right)\right)}$$

where

$$\begin{array}{ccc} Z & = & n{\bar{X}}_{1}+Y,\;c=k\left(n-1\right)/2\\ S^{2} & = & \sum_{i=1}^{k}\sum_{j=1}^{n}X_{ij}^{2}+Y^{2}\\ {\tilde{S}}^{2} & = & S^{2}-\left(n+1\right)\;{\left(\frac{Z}{n+1}\right)}^{2}-n\;\left(\sum_{i=2}^{k}{\bar{X}}_{i}^{2}\right)\\ r & = & \frac{\sqrt{n(n+1)}}{\tilde{S}}\left(\frac{Z}{n+1}-{\bar{X}}_{2}\right),\;\underline{r}=\min\left(r,1\right)\\ B(a,b) & = & \frac{\Gamma (a)\Gamma (b)}{\Gamma (a+b)}\\ & & F_{\beta (v_{1},v_{2})}\;\text{is}\;\text{the}\;\text{cdf}\;\text{of}\;\text{a}\;\text{Beta}\;\text{distribution}\;\text{with}\;\text{parameters}\;v_{1},v_{2}\text{.} \end{array}$$

Remark. Note that there are two errors in the formula for the UMVCUE as presented in Cohen and Sackrowitz (1989). Firstly, the summation in the definition of ${\tilde{S}}^{2}$ should start from *i* = 2, and not *i* = 1. In addition, the denominator of the second term of the UMVCUE should have a factor of 2^2*c*^, and not 2^*c* + 1^.

## 3. Extending the UMVCUE

A natural extension to the setting of Cohen and Sackrowitz is to consider *unequal* sample sizes. Suppose now that treatment *i* is tested on *n_i_* subjects in stage 1. The stage 1 data *X_ij_* are again normally distributed with mean μ_*i*_ and common unknown variance σ^2^. The stage 1 sample means ${\bar{X}}_{i}$ are given by ${\bar{X}}_{i}=\frac{1}{n_{i}}\sum_{j}^{n_{i}}X_{ij}$. We also now allow there to be more than one subject in stage 2.

Sometimes the properties of treatments that were not the highest ranked are of interest. Hence an additional extension is to find the UMVCUE of the mean of the *l*-th ranked treatment (*l* = 1, …, *k*). To this end, let ${\bar{Y}}_{l}$ be the mean of *m_l_* additional observations (denoted by *Y_lj_*) taken from the *l*-th ranked treatment group in stage 2.

Again conditioning on *Q*, we have the following UMVCUE for the mean μ_*l*_.

Theorem 3.1. The UMVCUE of μ_*l*_ given *Q* is

(2) $$\frac{Z_{l}}{n_{l}+m_{l}}-\sqrt{\frac{n_{l}}{m_{l}\left(n_{l}+m_{l}\right)}}\times \frac{{\tilde{S}}_{l}\left[{\left(1-{\underline{r}}_{l}^{2}\right)}^{c}-{\left(1-{\underline{q}}_{l}^{2}\right)}^{c}\right]}{2^{2c}cB\left(c,c\right)\left[F_{\beta (c,c)}\;\left(\frac{1}{2}\left({\underline{r}}_{l}+1\right)\right)-F_{\beta (c,c)}\;\left(\frac{1}{2}\left({\underline{q}}_{l}+1\right)\right)\right]}$$

where

$$\begin{array}{ccc} & & Z_{l}=n_{l}{\bar{X}}_{l}+m_{l}{\bar{Y}}_{l},\;c=\left(N-k\right)/2,\;N=\sum_{i=1}^{k}n_{i}\\ & & S_{l}^{2}=\sum_{i=1}^{k}\sum_{j=1}^{n_{i}}X_{ij}^{2}+m_{l}{\bar{Y}}_{l}^{2}\\ & & {\tilde{S}}_{l}^{2}=S_{l}^{2}-\left(n_{l}+m_{l}\right)\;{\left(\frac{Z_{l}}{n_{l}+m_{l}}\right)}^{2}-\sum_{i\neq l}n_{i}{\bar{X}}_{i}^{2}\\ & & r_{l}=\frac{\sqrt{n_{l}\left(n_{l}+m_{l}\right)/m_{l}}}{{\tilde{S}}_{l}}\left(\frac{Z_{l}}{n_{l}+m_{l}}-{\bar{X}}_{l+1}\right),\;{\underline{r}}_{l}=\min\left(r_{l},1\right)\;\text{for}\;\;l=1,\ldots ,k-1\\ & & q_{l}=\frac{\sqrt{n_{l}\left(n_{l}+m_{l}\right)/m_{l}}}{{\tilde{S}}_{l}}\left(\frac{Z_{l}}{n_{l}+m_{l}}-{\bar{X}}_{l-1}\right),\;{\underline{q}}_{l}=\max\left(q_{l},-1\right)\;\text{for}\;\;l=2,\ldots ,k \end{array}$$

and we define *q*_1_ = −1 and *r_k_* = 1.

Proof. The proof is similar to Cohen and Sackrowitz’s. We let

$$U_{l}=\sqrt{\frac{m_{l}\left(n_{l}+m_{l}\right)}{n_{l}}}\times \frac{{\bar{Y}}_{l}-\frac{Z_{l}}{n_{l}+m_{l}}}{{\tilde{S}}_{l}}.$$

The joint density of $\left(X_{ij},{\bar{Y}}_{l}\right)$ given *Q* is

$$\begin{array}{ccc} & & K\left(\mu ,\sigma^{2}\right)I_{Q}\left({\bar{x}}_{1},\ldots ,{\bar{x}}_{k}\right)\frac{1}{{\left(2\pi \sigma^{2}/m_{l}\right)}^{1/2}}\exp\;\left(-\frac{m_{l}}{2\sigma^{2}}{\left({\bar{y}}_{l}-\mu_{l}\right)}^{2}\right)\prod_{i,j}\frac{1}{{\left(2\pi \sigma^{2}\right)}^{1/2}}\\ & & \;\times \;\exp\;\left(-\frac{1}{2\sigma^{2}}{\left(x_{ij}-\mu_{i}\right)}^{2}\right)\\ & & \;\overset{\mu ,\sigma^{2}}{\propto }\;\exp\;\left(-\frac{1}{2\sigma^{2}}\left[\sum_{i,j}{\left(x_{ij}-\mu_{i}\right)}^{2}+m_{l}{\left({\bar{y}}_{l}-\mu_{l}\right)}^{2}\right]\right)\\ & & \;\overset{\mu ,\sigma^{2}}{\propto }\;\exp\;\left(-\frac{1}{2\sigma^{2}}\left[\left(\sum_{i,j}x_{ij}^{2}+m_{l}{\bar{y}}_{l}^{2}\right)-2\left(\sum_{j=1}^{n_{l}}x_{lj}+m_{l}y_{l}\right)\mu_{l}-2\sum_{i\neq l}\sum_{j=1}^{n_{i}}x_{ij}\mu_{i}\right]\right)\\ & & \;=\exp\;\left(-\frac{1}{2\sigma^{2}}\left[s_{l}^{2}-2z_{l}\mu_{l}-2\sum_{i\neq l}n_{i}{\bar{x}}_{i}\mu_{i}\right]\right) \end{array}$$

where $\;\propto^{\mu ,\sigma^{2}}\;$ indicates that we are ignoring terms proportional to μ or σ^2^, and $K\left(\mu ,\sigma^{2}\right)=E_{\mu ,\sigma^{2}}I_{Q}\left({\bar{x}}_{1},\ldots ,{\bar{x}}_{k}\right)$ with *I_Q_* denoting the indicator function of the ordering condition *Q*. Hence by the factorisation criterion, $\left(Z_{l},{\bar{X}}^{c},S_{l}^{2}\right)$ is a sufficient statistic, where ${\bar{X}}^{c}$ is the set of *k* − 1 variables $\left\{{\bar{X}}_{i},\;i=1,\ldots ,k,\;i\neq l\right\}$. The statistic is also complete, since it is from the exponential family of distributions (Lehmann and Romano 2005).

The UMVCUE of μ_*l*_ is the Rao-Blackwellisation of the unbiased estimator ${\bar{Y}}_{l}$, conditional on the complete, sufficient statistic $\left(Z_{l},{\bar{X}}^{c},S_{l}^{2}\right)$. That is, we seek the estimator $E({\bar{Y}}_{l}|Z_{l},{\bar{X}}^{c},S_{l}^{2},Q)$. Given the form of *U_l_*, showing that $E({\bar{Y}}_{l}|Z_{l},{\bar{X}}^{c},S_{l}^{2},Q)$ is equal to Equation (2) is equivalent to showing

(3) $$E\left(U_{l}|Z_{l},{\bar{X}}^{c},S_{l}^{2},Q\right)=-\frac{\left[{\left(1-{\underline{r}}_{l}^{2}\right)}^{c}-{\left(1-{\underline{q}}_{l}^{2}\right)}^{c}\right]}{2^{2c}cB\left(c,c\right)\left[F_{\beta (c,c)}\;\left(\frac{1}{2}\left({\underline{r}}_{l}+1\right)\right)-F_{\beta (c,c)}\;\left(\frac{1}{2}\left({\underline{q}}_{l}+1\right)\right)\right]}.$$

This in turn is equivalent to showing that the conditional density $f(u_{l}|z_{l},{\bar{x}}^{c},S_{l}^{2},Q)$ of $\left(U_{l}|Z_{l},{\bar{X}}^{c},S_{l}^{2},Q\right)$ is

(4) $$\frac{{\left(1-u^{2}\right)}^{c-1}}{\int_{{\underline{q}}_{l}}^{{\underline{r}}_{l}}{\left(1-u^{2}\right)}^{c-1}du}I_{Q^{'}}\left(u_{l},z_{l},{\bar{x}}^{c},S_{l}^{2}\right)$$

where $Q^{'}=\left\{{\underline{q}}_{l}<U_{l}<{\underline{r}}_{l}\;,\;{\bar{X}}_{1}>\cdots >{\bar{X}}_{l-1},\;{\bar{X}}_{l+1}>\cdots >{\bar{X}}_{k}\right\}$. This is because integrating $uf(u_{l}|z_{l},{\bar{x}}^{c},S_{l}^{2},Q)$ gives the right hand side of Equation (3).

Consider a new sampling model, where ${\bar{Y}}_{l}^{*}$ is the mean of *m_l_* additional observations from population *l*, without having observed ${\bar{X}}_{1}>{\bar{X}}_{2}>\cdots >{\bar{X}}_{k}$. Let S*_l_, Z_l_*, U*_l_ and S**_l_ be equal to *S_l_*, *Z_l_*, *U_l_* and ${\tilde{S}}_{l}$ with ${\bar{Y}}^{*}$ replacing $\bar{Y}$ in all formulae. Rearranging terms gives the following expression for U*_l_^2^:

(5) $${U_{l}^{*}}^{2}=\frac{\frac{m_{l}\left(n_{l}+m_{l}\right)}{n_{l}}{\left({\bar{Y}}_{l}^{*}-\frac{Z_{l}^{*}}{n_{l}+m_{l}}\right)}^{2}}{\frac{m_{l}\left(n_{l}+m_{l}\right)}{n_{l}}{\left({\bar{Y}}_{l}^{*}-\frac{Z_{l}^{*}}{n_{l}+m_{l}}\right)}^{2}+\sum_{i=1}^{k}\sum_{j=1}^{n_{i}}{\left(X_{ij}-{\bar{X}}_{i}\right)}^{2}}.$$

Hence U*_l_^2^ follows a $\beta (\frac{1}{2},c)$ distribution. Note that clearly U*_l_^2^ ⩽ 1⇒ − 1 ⩽ U_l_* ⩽ 1. Additional restrictions on U*_l_ come from conditioning on *Q*. Firstly, ${\bar{X}}_{l}>{\bar{X}}_{l+1}\Rightarrow {\bar{Y}}_{l}^{*}<\frac{1}{m_{l}}\left(Z_{l}^{*}-n_{l}{\bar{X}}_{l+1}\right)$. Hence U*_l_ < r_l_*, where

$$r_{l}^{*}=\frac{\sqrt{n_{l}\left(n_{l}+m_{l}\right)/m_{l}}}{S_{l}^{**}}\left(\frac{Z_{l}^{*}}{n_{l}+m_{l}}-{\bar{X}}_{l+1}\right).$$

Secondly, ${\bar{X}}_{l-1}>{\bar{X}}_{l}\Rightarrow {\bar{Y}}_{l}^{*}>\frac{1}{m_{l}}\left(Z_{l}^{*}-n_{l}{\bar{X}}_{l-1}\right)$. Hence U*_l_ > q_l_*, where

$$q_{l}^{*}=\frac{\sqrt{n_{l}\left(n_{l}+m_{l}\right)/m_{l}}}{S_{l}^{**}}\left(\frac{Z_{l}^{*}}{n_{l}+m_{l}}-{\bar{X}}_{l-1}\right).$$

Note that U*_l_ is an ancillary statistic, and so by Basu's theorem (Basu 1955) is independent of $Z_{l}^{*},{\bar{X}}^{c},{S_{l}^{*}}^{2}$. Hence the joint density of $\left(U_{l}^{*},Z_{l}^{*},{\bar{X}}^{c},{S_{l}^{*}}^{2}\right)$ is

(6) $$g(u_{l}^{*})h(z_{l}^{*},{\bar{x}}^{c},{S_{l}^{*}}^{2})$$

where *g*(u*_l_) is the density of U*_l_ and $h(z_{l}^{*},{\bar{x}}^{c},{S_{l}^{*}}^{2})$ is the density of $\left(Z_{l}^{*},{\bar{X}}^{c},{S_{l}^{*}}^{2}\right)$. The numerator of the conditional density of $U_{l}^{*}|\left(Z_{l}^{*},{\bar{X}}^{c},{S_{l}^{*}}^{2},Q\right)$ is then Equation (6) times

$$K^{-1}\left(\mu ,\sigma^{2}\right)I_{Q*}(u_{l}^{*},z_{l}^{*},{\bar{x}}^{c},{S_{l}^{*}}^{2})$$

where

$$\begin{array}{ccc} & & Q^{*}=\left\{{\underline{q}}_{l}^{*}<U_{l}^{*}<{\underline{r}}_{l}^{*},\;{\bar{X}}_{1}>\cdots >{\bar{X}}_{l-1},\;{\bar{X}}_{l+1}>\cdots >{\bar{X}}_{k}\right\},\\ & & {\underline{r}}_{l}^{*}=\min\left(r_{l}^{*},1\right),\;{\underline{q}}_{l}^{*}=\max\left(q_{l}^{*},-1\right), \end{array}$$

with *I*_Q*_ being the indicator functions of the ordering condition Q*.

The denominator of the conditional density of $U_{l}^{*}|\left(Z_{l}^{*},{\bar{X}}^{c},{S_{l}^{*}}^{2},Q\right)$ is the integral of Equation (6) with respect to u*_l_ from ${\underline{q}}_{l}^{*}$ to ${\underline{r}}_{l}^{*}$. This can be calculated using the fact that U*_l_^2^ follows a $\beta (\frac{1}{2},c)$ distribution. Putting everything together gives the conditional density equivalent to Equation (4).

Remark 1. As an important special case, setting *l* = 1 in Equation (2) gives the following UMVCUE for the mean of the highest-ranking treatment:

(7) $$\frac{Z_{1}}{n_{1}+m_{1}}-\sqrt{\frac{n_{1}}{m(n_{1}+m_{1})}}\times \frac{\tilde{S}{\left(1-{\underline{r}}^{2}\right)}^{c}}{2^{2c}cB\left(c,c\right)F_{\beta (c,c)}\;\left(\frac{1}{2}\left(\underline{r}+1\right)\right)}$$

where

$$\begin{array}{ccc} & & Z_{1}=n_{1}{\bar{X}}_{1}+m_{1}{\bar{Y}}_{1},\;S^{2}=\sum_{i=1}^{k}\sum_{j=1}^{n_{i}}X_{ij}^{2}+m_{1}{\bar{Y}}_{1}^{2}\\ & & {\tilde{S}}^{2}=S^{2}-\left(n_{1}+m_{1}\right)\;{\left(\frac{Z}{n_{1}+m_{1}}\right)}^{2}-\sum_{i=2}^{k}n_{i}{\bar{X}}_{i}^{2}\\ & & r=\frac{\sqrt{n_{1}\left(n_{1}+m_{1}\right)/m_{1}}}{\tilde{S}}\left(\frac{Z_{1}}{n_{1}+m_{1}}-{\bar{X}}_{2}\right),\;\underline{r}=\min\left(r,1\right) \end{array}$$

Remark 2. As a simple check of consistency with Section 2, set *n_i_* = *n* (for *i* = 1, …, *k*) and *m*_1_ = 1 in Equation (7). Then we recover the corrected Equation (1) of Cohen and Sackrowitz.

## 4. Comparison with the known variance setting

Assuming a common known variance σ^2^, we can use the results of Bowden and Glimm (2008) to find the UMVCUE. Letting ϕ and Φ denote the pdf and cdf respectively of a standard normal distribution, the UMVCUE of μ_*l*_ is as follows.

Theorem 4.1. The UMVCUE of μ_*l*_ given *Q* is

(8) $$\frac{Z_{l}}{n_{l}+m_{l}}-\sqrt{\frac{n_{l}}{m_{l}\left(n_{l}+m_{l}\right)}}\times \frac{\sigma [\varphi \left(W_{l,l+1}\right)-\varphi \left(W_{l,l-1}\right)]}{\left[\Phi \left(W_{l,l+1}\right)-\Phi \left(W_{l,l-1}\right)\right]}$$

where

$$\begin{array}{ccc} & & W_{l,l+1}=\frac{\sqrt{n_{l}\left(n_{l}+m_{l}\right)/m_{l}}}{\sigma }\left(\frac{Z_{l}}{n_{l}+m_{l}}-{\bar{X}}_{l+1}\right)\\ & & W_{l,l-1}=\frac{\sqrt{n_{l}\left(n_{l}+m_{l}\right)/m_{l}}}{\sigma }\left(\frac{Z_{l}}{n_{l}+m_{l}}-{\bar{X}}_{l-1}\right) \end{array}$$

and we define *X*_0_ = ∞ and *X*_*k* + 1_ = −∞.

Remark. This is structurally the same as the UMVCUE with unknown variances given by Equation (2). Firstly, both estimators are in the form of the MLE $\frac{Z_{l}}{n_{l}+m_{l}}$ minus a bias correction term, where the latter has a multiplicative factor of $\sqrt{\frac{n_{l}}{m_{l}\left(n_{l}+m_{l}\right)}}$.

The role of the known standard deviation σ in Equation (8) is played by the estimate ${\tilde{S}}_{l}$ in Equation (2). Hence *r_l_* and *q_l_* in Equation (2) can be seen to be exact analogues of *W*_*l*, *l* + 1_ and  *W*_*l*, *l* − 1_ respectively in Equation (8). Finally, the standard normal density and distribution functions in Equation (8) are replaced by (transformed) beta density and distribution functions in Equation (2).

### 4.1. Simulation study

We now conduct simulation studies to assess how the UMVCUE with a known variance performs when the assumed known variance ${\hat{\sigma }}^{2}$ is not exactly equal to the true variance σ^2^. We compare various estimators for the largest mean μ_1_:

- Stage 2 estimator, which is independent of σ^2^.
- MLE, which is independent of σ^2^.
- ${\hat{U}}_{\text{known}}$, the UMVCUE which assumes that σ^2^ is known, and equal to some value ${\hat{\sigma }}^{2}$.
- ${\hat{U}}_{\text{unknown}}$, the UMVCUE with unknown σ^2^.

To evaluate the performance of a generic estimator for μ_1_, say μ*_1_, we use the following definitions of the bias and MSE, as in Bowden and Glimm (2008):

$$\begin{array}{ccc} b_{\text{sel}}\left(\mu_{1}^{*}\right) & = & \sum_{i=1}^{K}E\left[\mu_{1}^{*}-\mu_{i}|X_{1}=X_{i}\right]P\left(X_{1}=X_{i}\right),\\ {\text{MSE}}_{\text{sel}}\left(\mu_{1}^{*}\right) & = & \sum_{i=1}^{K}E\left[{\left(\mu_{1}^{*}-\mu_{i}\right)}^{2}|X_{1}=X_{i}\right]P\left(X_{1}=X_{i}\right). \end{array}$$

We first compare characteristics of the estimators for trials with different target powers. Consider testing the hypothesis *H*_0_: μ_1_ ⩽ 0 against the alternative *H*_1_: μ_1_ > 0 at the end of the trial. To do so, we use the usual *t*-statistic

$$T_{d}=\frac{Z_{1}}{\hat{\sigma }\sqrt{n_{1}+m_{1}}}$$

where ${\hat{\sigma }}^{2}$ is the pooled sample variance of the stage 1 and stage 2 data, i.e.

(9) $${\hat{\sigma }}^{2}=\frac{1}{N+m_{1}-k-1}\left(\sum_{i=1}^{k}\sum_{j=1}^{n_{i}}{\left(X_{ij}-{\bar{X}}_{i}\right)}^{2}+\sum_{j=1}^{m_{1}}{\left(Y_{1j}-{\bar{Y}}_{1}\right)}^{2}\right).$$

Under *H*_0_, *T_d_* follows a *t*-distribution with *d* = *N* + *m*_1_ − *k* − 1 degrees of freedom. Hence, when testing at significance level α, we reject *H*_0_ if *T_d_* > *t* ^− 1^_*d*_(1 − α), where *t* ^− 1^_*d*_ denotes the inverse cdf of a *t*-distribution with *d* degrees of freedom.

Suppose we are targeting a power of at least (1 − β) to detect a λ–fold standard deviation increase in the mean, i.e. under the alternative hypothesis $H_{1}:\mu_{1}=\lambda \hat{\sigma }$. This implies that

(10) $$n_{1}+m_{1}\geq \frac{1}{\lambda }{\left[t_{d}^{-1}\left(1-\alpha \right)+t_{d}^{-1}\left(1-\beta \right)\right]}^{2}.$$

For simplicity, suppose we have equal numbers of subjects for each treatment and each stage, denoted by *M*, so that *n*_1_ = *m*_1_ = *M*, *N* = *kM* and hence *d* = *M*(*k* + 1) − *k* − 1. For given values of α, β and λ, Equation (10) can be solved for *M* numerically.

In our simulation study, α = 0.05 and we vary β from 0.05 to 0.5, which corresponds to trials with powers between 50% to 95%. We set *k* = 3, with μ = (0, 0, 0), σ^2^ = 1 and λ = 0.5. For the estimator ${\hat{U}}_{\text{known}}$, we calculate ${\hat{\sigma }}^{2}$ as the pooled sample variance given in Equation (9) and simply plug it in to Equation (8).

**Figure 1.**
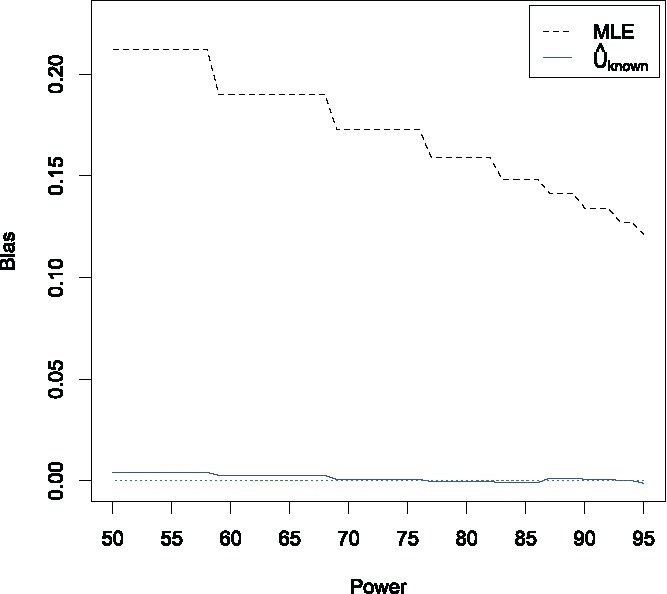
Mean bias of the MLE and ${\hat{U}}_{\text{known}}$ as the power varies from 50% to 95%. Results are based on 10^5^ simulations for each value of the per-group sample size *M*.

Figure 1 shows the mean bias of the MLE and ${\hat{U}}_{\text{known}}$ as the power varies from 50% (which corresponds to *M* = 4) to 95% (which corresponds to *M* = 12). We also simulated the bias of ${\hat{U}}_{\text{unknown}}$ and the stage 2 estimator, but as expected, this resulted in no bias apart from simulation error. Hence these simulation results are not shown here, and we simply plot where the bias equal to zero. The MLE is positively biased, with a mean bias ranging from 0.21 (for a power between 50% and 58%) to 0.12 (for a power of 95%). In contrast, ${\hat{U}}_{\text{known}}$ is essentially unbiased, although there seems to be a very small positive bias when the power is less than 58%.

**Figure 2.**
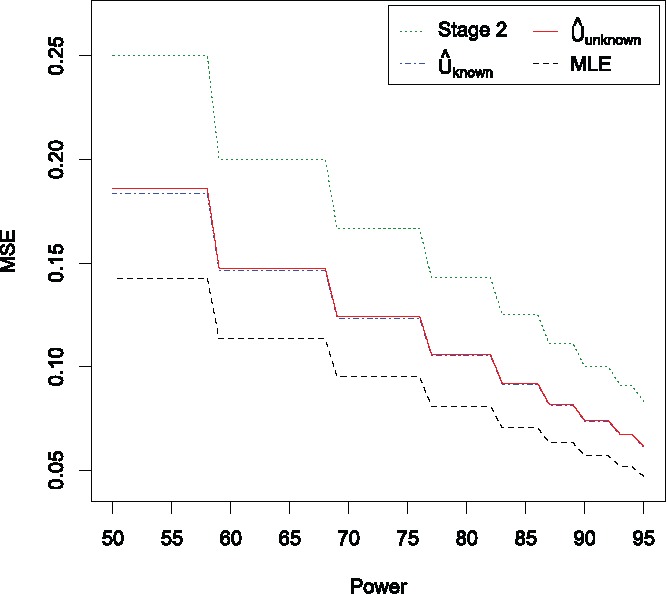
MSE of the four estimators as the power varies from 50% to 95%. Results are based on 10^5^ simulations for each value of the sample size *M*.

Figure 2 shows the MSE of the four estimators. The stage 2 estimator has the highest MSE, while the MLE has the lowest. The estimators ${\hat{U}}_{\text{unknown}}$ and ${\hat{U}}_{\text{known}}$ have approximately the same MSE, which is 29%–30% higher than the MSE for the MLE, but 26%–27% lower than the MSE for the stage 2 estimator.

**Figure 3.**
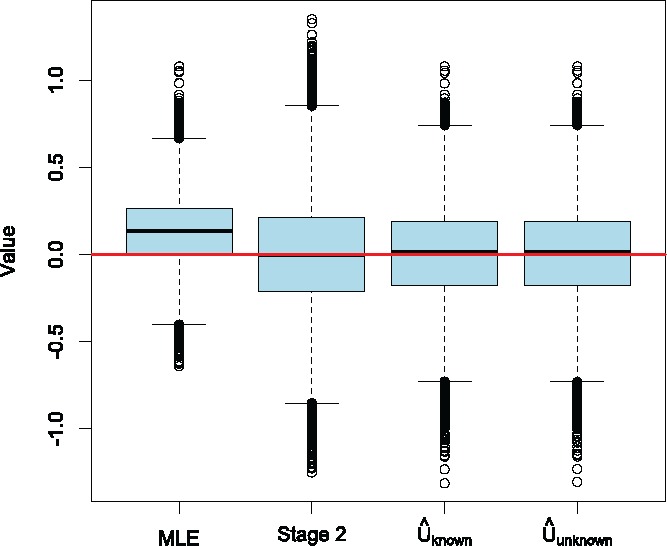
Boxplots of the four estimators for a sample size *M* = 10, based on 10^5^ simulations.

To demonstrate the differences further, Figure 3 shows the boxplots for the estimators when *M* = 10, which corresponds to a power of 90%. The boxplots demonstrate the severity of the positive bias of the MLE, and the unbiasedness of the other three estimators. The distribution of the estimators ${\hat{U}}_{\text{unknown}}$ and ${\hat{U}}_{\text{known}}$ seem to be almost identical.

To explore what sort of estimates of $\hat{\sigma }$ lead to substantial differences between ${\hat{U}}_{\text{unknown}}$ and ${\hat{U}}_{\text{known}}$, we conduct a further simulation study which does not assume that we are using the pooled sample variance as our estimate of $\hat{\sigma }$. Keeping *M* = 10 as before, we vary the value of ${\hat{\sigma }}^{2}$ from 0.25 to 4 and simply plug it in to Equation (8). In order to get a handle on the likely values of ${\hat{\sigma }}^{2}$ that could be estimated from the data, note that ${\hat{\sigma }}^{2}∼\frac{\sigma^{2}}{d}\chi_{d}^{2}$, and hence $\text{var}\left({\hat{\sigma }}^{2}\right)=\frac{2\sigma^{2}}{d}$.

For *M* = 10, σ^2^ = 1 and *k* = 3, this gives $\text{var}({\hat{\sigma }}^{2})\approx 0.0556$. Hence in our simulation setting, a very simple approximate 95% confidence interval for ${\hat{\sigma }}^{2}$ is given by $1\pm 2\times \sqrt{0.0556}$, which we show on the plots below as a shaded gray region.

**Figure 4.**
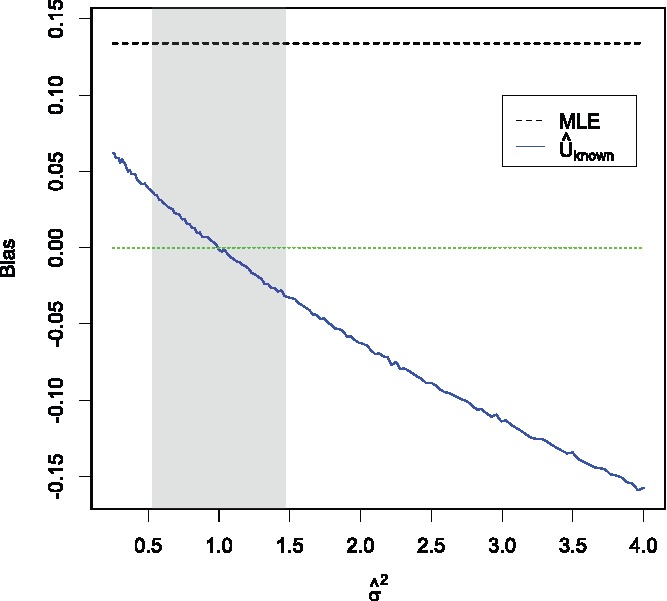
Mean bias of the MLE and ${\hat{U}}_{\text{known}}$ as ${\hat{\sigma }}^{2}$ varies from 0.25 to 4. Results are based on 10^5^ simulations for each value of ${\hat{\sigma }}^{2}$. The gray region shows an approximate 95% confidence interval for ${\hat{\sigma }}^{2}$.

Figure 4 shows the mean bias of the MLE and ${\hat{U}}_{\text{known}}$. The MLE has a positive bias of $\frac{3}{4\sqrt{10\pi }}=0.1338...$ for all values of ${\hat{\sigma }}^{2}$. In contrast ${\hat{U}}_{\text{known}}$ is positively biased for ${\hat{\sigma }}^{2}<1$ and negatively biased for ${\hat{\sigma }}^{2}>1$. The symmetry of the positive and negative bias of ${\hat{U}}_{\text{known}}$ around ${\hat{\sigma }}^{2}=1$ explains why, on average, using a pooled sample variance estimator (which would be expected to under and overestimate σ^2^ almost equally) results in an essentially unbiased estimator.

**Figure 5.**
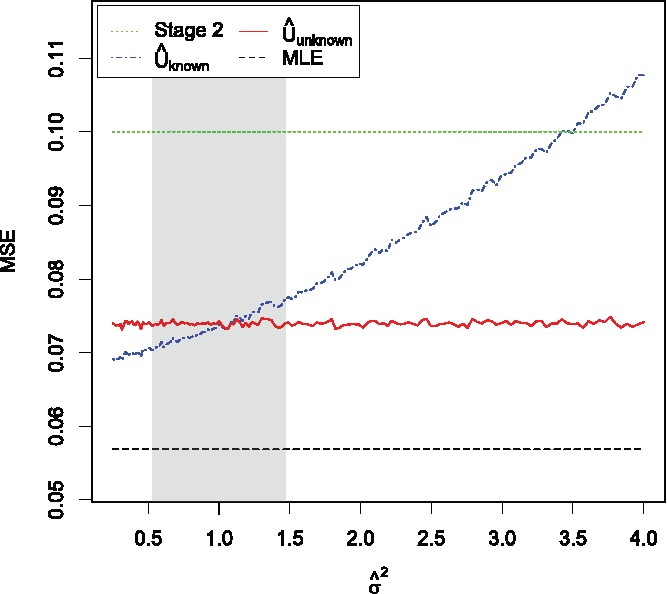
MSE of the four estimators as ${\hat{\sigma }}^{2}$ varies from 0.25 to 4. Results are based on 10^5^ simulations for each value of ${\hat{\sigma }}^{2}$. The gray region shows an approximate 95% confidence interval for ${\hat{\sigma }}^{2}$.

Figure 5 shows the MSE of the four estimators. The stage 2 estimator has the highest MSE of 0.1, while the MLE has the lowest MSE of $\frac{1}{20}\left(1+\frac{\sqrt{3}}{4\pi }\right)=0.05689...$ for all values of ${\hat{\sigma }}^{2}$. The estimator ${\hat{U}}_{\text{unknown}}$ has a MSE of approximately 0.074. This is a 26% decrease compared to the stage 2 estimator, but a 43% increase compared to the MLE. Finally, ${\hat{U}}_{\text{known}}$ has a lower MSE than ${\hat{U}}_{\text{unknown}}$ for ${\hat{\sigma }}^{2}<1$, which can be explained by regarding ${\hat{U}}_{\text{known}}$ as a sort of shrinkage estimator. Again, the symmetry of the difference between the MSEs of ${\hat{U}}_{\text{known}}$ and ${\hat{U}}_{\text{unknown}}$ around ${\hat{\sigma }}^{2}=1$ explains why, on average, the MSEs of these two estimators are so similar.

## 5. Case study

Finally, we illustrate our methodology using data based on the INHANCE study (Lawrence, Bretz, and Pocock 2014), which evaluated the use of inhaled indacaterol for the treatment of patients suffering from chronic obstructive pulmonary disease (COPD). The study followed a two-stage adaptive seamless design, where the first stage was a dose-finding stage with dose selection at the interim analysis, and the second stage was a confirmatory analysis of the efficacy and safety of the selected doses. The primary outcome was the 24-hour post-dose ${\text{FEV}}_{1}$ (which is the Forced Expiratory Volume after one second).

In the study, four doses of indacaterol were tested in stage 1 (75 μg, 150 μg, 300 μg and 600 μg), and two doses were selected based on a set of dose selection guidelines (see pages 84–87 in Lawrence, Bretz, and Pocock (2014) for further details). For simplicity, and in order for the selected doses to be the same as those that would be selected when ranking by sample means, we only consider the three doses 75 μg, 150 μg and 300 μg of indacaterol.

Table 1 gives the observed least-squares mean treatment difference versus placebo at week 2 for each dose of indacaterol for the two stages, with the stage 1 results as given in Barnes et al. (2010), and the stage 2 results as given in Donohue et al. (2010). On the basis of ranking by the sample means, doses 150 μg and 300 μg would be selected for confirmatory analysis in stage 2.

**Table 1.** Sample sizes used for the case study, with the observed treatment difference from placebo at week 2 as observed in the INHANCE study.

	**Stage 1**		**Stage 2**	
Indacaterol dose (μg)	*n_i_*	Treatment difference vs placebo (L)	*m_i_*	Treatment difference vs placebo (L)
75	10	0.15	—	—
150	9	0.18	9	0.18
300	7	0.21	9	0.18

The INHANCE study had over 100 patients randomised to each of the three doses in stage 1, and over 400 patients randomised to each of the two selected doses in stage 2. With these large sample sizes, we would not expect there to be appreciable differences between the estimators. Hence, for the purposes of our illustrative case study, we consider a trial with only *n* = (10, 9, 7) patients in stage 1, and 9 patients for each dose in stage 2, as shown in Table 1.

We use the observed mean treatment differences from each stage of the INHANCE study to simulate a realisation of the data with the sample sizes given above. For each dose at each stage, the data was simulated from a normal distribution with mean equal to that observed in the INHANCE study, and a standard deviation of 0.3L (as given in the results of Donohue et al. 2010).

Table 2 shows the various estimators for the treatment difference from placebo (L) at week 2 for the two selected doses. Note that for ${\hat{U}}_{\text{known}}$, we use the pooled sample variance as given in Equation (9) as our estimate for ${\hat{\sigma }}^{2}$. For both doses, there is a drop in the mean between the stage 1 and stage 2 data, with the MLE in-between since it is a weighted mean. For the 300 μg dose, there are only very small differences between the MLE, ${\hat{U}}_{\text{known}}$ and ${\hat{U}}_{\text{unknown}}$, while for the 150 μg dose these three estimators are in fact identical. Hence for these data, there would be no practical difference in using any of these three estimators to correct for selection bias.

**Table 2.** Estimators for the treatment difference from placebo (L) at week 2, based on results from the INHANCE study.

Indacaterol dose (μg)	Stage 1	Stage 2	MLE	${\hat{U}}_{\text{known}}$	${\hat{U}}_{\text{unknown}}$
300	0.218	0.179	0.196	0.194	0.195
150	0.192	0.180	0.186	0.186	0.186

## 6. Discussion

In two-stage adaptive trials with normally distributed data, it is unlikely that the variance will be known exactly. Our modified estimator allows for efficient unbiased estimation of multiple selected treatments with a common unknown variance, where the stage one and two sample sizes are arbitrary.

As the simulation studies demonstrated, when using the pooled sample variance, on average the UMVCUE ${\hat{U}}_{\text{known}}$ that assumes a known variance will be essentially unbiased, with a very similar MSE to our modified UMVCUE. However, if the variance is under or overestimated, then ${\hat{U}}_{\text{known}}$ is no longer unbiased, with a higher MSE if the variance is overestimated. We note that our proposed estimator is most useful when the sample sizes are small. Indeed, if ${\hat{\sigma }}^{2}$ is estimated using the pooled sample variance, then for *d* > 200, $\text{var}\left({\hat{\sigma }}^{2}\right)<0.01\sigma^{2}$ and there is unlikely to be any substantial variation of ${\hat{\sigma }}^{2}$ from the true value σ^2^.

In this paper, we only looked at ranking by the treatment means. However, it should be reasonably straightforward to adapt our result to ranking by other criteria. One example would be ranking by one-sided ‘*p*-value’, where the conditioning would change to the event $Q^{'}=\left\{X:\sqrt{n_{1}}{\bar{X}}_{1}>\cdots >\sqrt{n_{k}}{\bar{X}}_{k}\right\}$. Then the proof follows through identically to give the formula (2), except that the ${\bar{X}}_{l+1}$ in *r_l_* is replaced by $\sqrt{n_{l+1}}{\bar{X}}_{l+1}/\sqrt{n_{l}}$, and the ${\bar{X}}_{l-1}$ in *q_l_* is replaced by $\sqrt{n_{l-1}}{\bar{X}}_{l-1}/\sqrt{n_{l}}$.

The proof of the UMVCUE is quite a delicate one, and we do not see a straightforward way to generalise the result to the setting where we no longer assume a *common* unknown variance but instead let treatment *i* have variance σ^2^_*i*_ say. The problem is that U*_l_^2^ as given in Equation (5) is no longer a ratio of independent chi-squared distributions, and would now depend on the unknown parameters σ^2^_1_, …, σ_*k*_^2^.

Finally, the focus of this paper was on point estimation, but it is natural to try to derive confidence intervals for the UMVCUE as well. One approach is to use a bootstrap procedure, similar to that described in e.g., Bowden and Glimm (2008) and Robertson, Prevost, and Bowden (2016a). Alternatively, the approach of Sampson and Sill (2005) can possibly be extended to give exact confidence intervals in the unknown variance setting.
